# Detection and quantification of γ-H2AX using a dissociation enhanced lanthanide fluorescence immunoassay

**DOI:** 10.1038/s41598-021-88296-3

**Published:** 2021-04-26

**Authors:** Felicite K. Noubissi, Amber A. McBride, Hannah G. Leppert, Larry J. Millet, Xiaofei Wang, Sandra M. Davern

**Affiliations:** 1 Department of Biology, Jackson State University, Jackson, MS USA; 2 Biosciences Division, Oak Ridge National Laboratory, Oak Ridge, TN USA; 3 Center for Environmental Biotechnology, University of Tennessee, Knoxville, TN USA; 4 Department of Biological Sciences, Tennessee State University, Nashville, TN USA; 5 Radioisotope Science and Technology Division, Oak Ridge National Laboratory, Oak Ridge, TN USA

**Keywords:** Biochemistry, Biological techniques, Cancer

## Abstract

Phosphorylation of the histone protein H2AX to form γ-H2AX foci directly represents DNA double-strand break formation. Traditional γ-H2AX detection involves counting individual foci within individual nuclei. The novelty of this work is the application of a time-resolved fluorescence assay using dissociation-enhanced lanthanide fluorescence immunoassay for quantitative measurements of γ-H2AX. For comparison, standard fluorescence detection was employed and analyzed either by bulk fluorescent measurements or by direct foci counting using BioTek Spot Count algorithm and Gen 5 software. Etoposide induced DNA damage in A549 carcinoma cells was compared across all test platforms. Time resolved fluorescence detection of europium as a chelated complex enabled quantitative measurement of γ-H2AX foci with nanomolar resolution. Comparative bulk fluorescent signals achieved only micromolar sensitivity. Lanthanide based immunodetection of γ-H2AX offers superior detection and a user-friendly workflow. These approaches have the potential to improve screening of compounds that either enhance DNA damage or protect against its deleterious effects.

## Introduction

DNA damage in cells can be triggered by endogenous mechanisms, exogenous factors, or a combination of both, resulting in a multitude of alterations including DNA base modifications, single-strand breaks, and double-strand breaks (DSBs)^1^. DNA DSBs present the most serious threat to the cell. They are difficult to repair and can lead to genotoxicity, cell death, and misrepaired DNA DSBs with potential for neoplastic progression. Exogenous factors inducing DNA DSBs include cytotoxic chemical agents^2–4^ and environmental^5^ and physical factors such as radiation^6–9^ and heat^10,11^. Recent studies using a very fast CRISPR Cas 9 system to induce DNA DSBs have tracked their formation over time and length scales^12^. The ability of different forms of irradiation to induce DNA DSBs has been harnessed in radiotherapy treatments to kill cancer cells^13,14^. However, given the detrimental effects of DNA DSBs on cell viability and the induction of mutated cells, it is critical to quantify them effectively to better inform treatment decisions. Effective and efficient quantification of DSBs is critical for managing the effective dose for radiation therapy, identifying exposure to cytotoxic agents or environmental factors, and assessing the pharmacodynamics of chemotherapeutics. In addition, manual or semi-automated assessment of DSBs can be used to elucidate the protective effect of compounds against genomic damage^15^, assess the DNA repair process of cells^1^, or even establish the endogenous load of DNA DSBs within the cells^16^.

**Figure 1:**
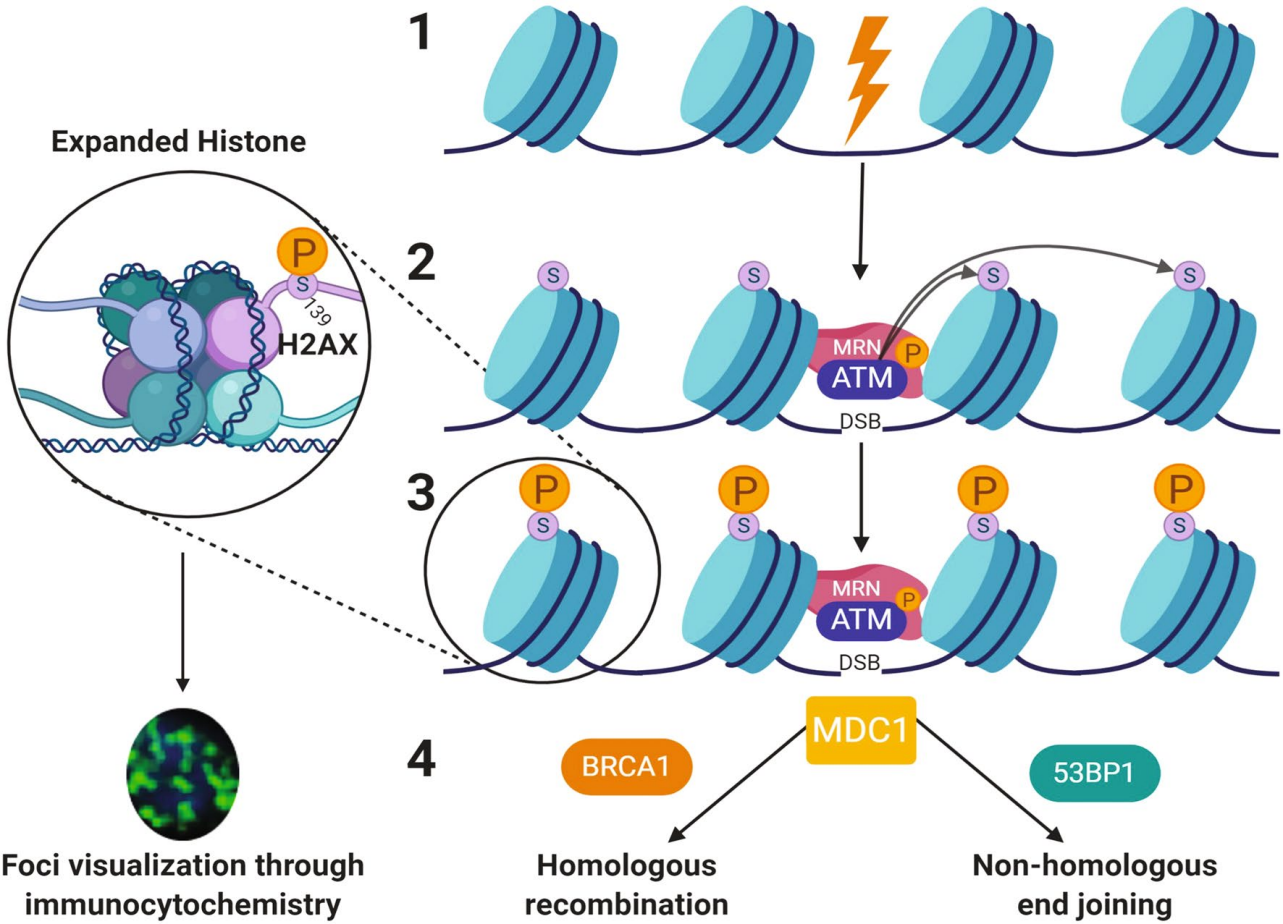
DNA damage and repair pathway. (1) Mutagens (ionizing radiation, chemotherapeutics, or clastogens) induce double-strand breaks in DNA-wrapped histones. (2) Breaks in the DNA double-strand recruit the MRN complex composed of MRe11, Rad50, and Nbs1 proteins, which then recruit and activate ATM kinase. (3) ATM kinase phosphorylates the H2AX histone protein on the serine 139 residue (expanded histone) to create phosphorylated foci that can be visualized through the γ-H2AX assay and immunocytochemistry. (4) The mediator of DNA damage checkpoint protein 1 (MDC1) is recruited to the DSB. After modification via ATM, MDC1 recruit proteins, such as BRCA1 and 53BP1, to direct the DNA damage and repair the pathway through homologous recombination or nonhomologous end-joining respectively. Image created with BioRender.com, https://biorender.com/.

DNA DSBs trigger a DNA damage response that is characterized by activation of DNA repair mechanisms and phosphorylation of the histone protein component H2AX to form phosphorylated H2AX (γ-H2AX)^17–20^. H2AX is one of the most conserved variants of the histone H2A and accounts for 2–25% of the H2A protein^21^. H2AX becomes phosphorylated on its residue serine 139 in cells when DNA DSBs occur^18^. This phosphorylation is performed by members of the phosphatidylinositol-3-OH-kinase-like family of protein kinases, including ataxia telangiectasia mutated (ATM), ATM-Rad3-related, and DNA-dependent protein kinase^22,23^. The initial phosphorylation of H2AX is followed by a sequential recruitment of MDC1 (mediator of DNA damage checkpoint) and MRN (NBS1/hMRE11/hRAD50) repair complex resulting in further activation of ATM and subsequent phosphorylation of hundreds to thousands of H2AX histone proteins in large chromosomal domains surrounding the DSBs (Fig. 1).

The γ-H2AX interacts with hundreds of other proteins and protein complexes such as MDC1, MRN, 53BP1, and BRCA1/BARD1 to form foci in the region of DSBs^24,25^. The γ-H2AX foci have been shown to represent a good approximation of DNA DSBs and have been therefore established as a marker for DSBs^26,27^. Since γ-H2AX may also be associated with non-DSB related DNA damage it is important to note the contribution of these factors^16,28^. Immunofluorescence techniques to quantify γ-H2AX have been developed as a surrogate marker for DNA DSB quantification^29^. It is clear that these methods also reveal clustered DNA damage which are more difficult to repair and potentially more mutagenic^30^. The dynamics of γ-H2AX formation and spreading at DSB foci have recently been tracked showing expansion with time at the cleavage site to encompass up to 11 megabases of DNA, which may indicate variation in foci size and duration change during DNA repair^30^. Other methods developed to measure DNA DSBs include neutral elution^31^, pulsed-field electrophoresis^32,33^, and the comet assay^34,35^. More recently, qPCR^28^, breaks labeling in situ with sequencing (BLISS)^29^, and qDSB-Seq^30^ have also been used. However, direct quantification of γ-H2AX is simpler, more convenient, and a more sensitive measure than many of the above-mentioned methods. Importantly, it does not require lysing the cells, a process needed for many other assays.

The use of fluorescence has been applied to different assays to quantify γ-H2AX^36^. This includes epifluorescence microscopy^4^, confocal microscopy, and 3D reconstructed images^37^, western blot^22^, and flow cytometry^38^. These methods are often less sensitive, tedious, and require manual efforts to count γ-H2AX foci using an epifluorescent microscope. Manual labor to count γ-H2AX foci in cells is a low throughput process, but has trade-offs in accuracy and statistical representation, and may have the potential for reviewer-to-reviewer discrepancy or bias^20,39^. A variety of robust high-throughput methods such as laser-scanning cytometry^3^, infrared imaging scanners^5^, imaging flow cytometry^40^, automated γ-H2AX immunocytochemistry^41^, and autofoci^42^ have been established to quantify γ-H2AX in cells. Most of these systems are automated and designed to detect γ-H2AX in large-scale studies and are therefore suitable for laboratories specialized in handling large amounts of samples. Although they offer many advantages in terms of sensitivity and reduced experimental time, these methods are often sample specific and require sophisticated instrumentation or costly technology. While, automated imaging instrumentation and image processing programs allow for semi-automated data acquisition, these systems may have increased errors in γ-H2AX events^39^ (e.g., overlapping or clustered foci^43^).

In this study, we have developed methods for highly automated***,*** multimodal detection of γ-H2AX foci at a range of expression levels to allow quantification of DNA DSBs in cells. Our methodology is based on the use of commonly available plate readers capable of fluorescence and time-resolved fluorescence (TRF) measurements. We have quantified DNA DSBs by measuring the total fluorescence intensity of γ-H2AX labeled with the fluorophore Alexa Fluor 488 for analysis in a 96-well plate format using a plate reader. Concurrently, we quantified γ-H2AX using a lanthanide-chelated secondary antibody to perform a dissociation-enhanced lanthanide fluorescence immunoassay (DELFIA)^44^ which employs TRF intensity technology and provides a high sensitivity (signal to background) detection that can be used to quantify low (femtogram^44^) levels of DNA DSBs within cells more accurately. We compared our findings to more traditional foci counting methodology by quantifying the labeled γ-H2AX through spot counting using a Spot Count algorithm used in conjunction with the BioTek Cytation 1. This method is comparable to the DELFIA assay in its dynamic range and exhibits a more sensitive detection of γ-H2AX foci than the assay employing direct fluorescence measurement of an Alexa Fluor 488 labelled antibody. The use of DELFIA for γ-H2AX detection may be of benefit beyond the automated assays where single foci are enumerated, given that it does not need to distinguish between foci of different size. This assay offers the advantage that it does not determine single foci from clusters of foci, but rather relies on quantification of γ-H2AX fluorescence intensity in lieu of foci count.

The multimodal detection of γ-H2AX developed here offers further methods to quantify γ-H2AX that could be used to monitor and improve both radiotherapy and chemotherapy and would be particularly useful to screen compound libraries to find potential radiation or chemotherapy mitigation or sensitization agents. Both the DELFIA and the Spot Count algorithm assays have the potential to sensitively detect low level alterations in DNA damage induced by environmental chemical or physical factors; therefore, these assays are expected to add to the repertoire of analytical techniques that can be used to advance studies in this field.

## Results

### Quantification of γ-H2AX fluorescence intensity as a function of cell density

**Figure 2:**
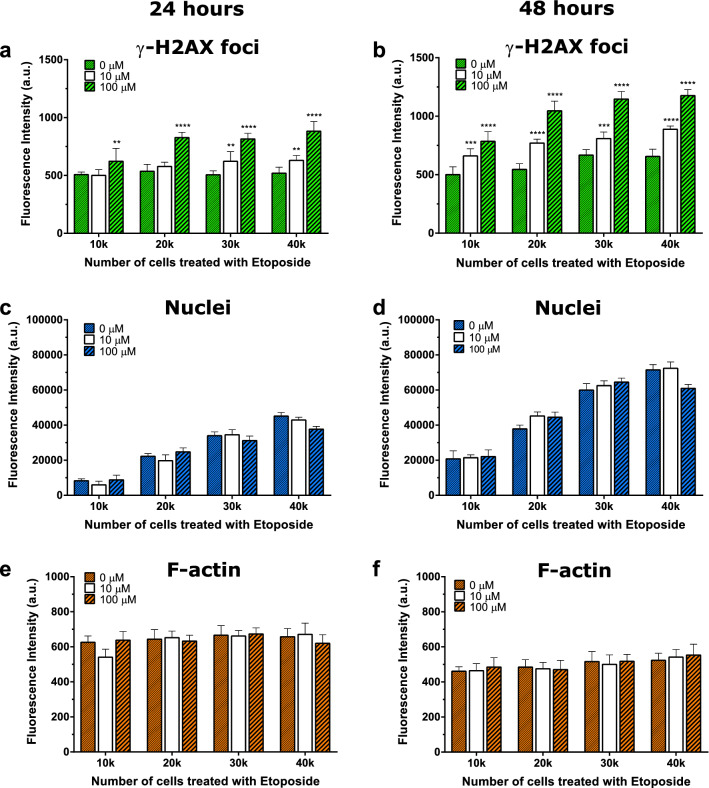
Nuclear γ-H2AX bulk fluorescence intensity detected using a plate reader as a function of cell density. Cells plated at increasing cell densities 10,000, 20,000, 30,000, and 40,000 per well (320 mm^2^, 96 well plate), grown for either 24 h (panels **a**,**c**, and **e**) or 48 h (panels **b**,**d**, and **f**) were treated with etoposide (0, 10, 100 μM). (**a**,**b**) Cells immunolabeled for γ-H2AX phosphorylation to quantify DNA double-strand breaks. (**c**,**d**) Genomic DNA was labeled (DAPI) to identify the cellular nuclei for γ-H2AX co-localization and cell density measures per well. (**e**,**f**) Rhodamine–phalloidin labeled the F-actin for cell density measures. N = 6 wells per treatment, data are presented as mean ± SEM. **P < 0.01. ***P < 0.001, ****P < 0.0001 (compared with 0 µM etoposide), one-way ANOVA followed by Tukey multiple comparisons post-test.

A preliminary form of this work was presented in a poster session at The Society for Nuclear Medicine and Molecular Imaging annual meeting^45^. We assessed the impact of cell density and growth on the identification of DSBs using γ-H2AX total fluorescence detection. Quantification of fluorescently labeled DNA DSBs was assessed for A549 lung epithelial carcinoma cells exposed to etoposide (0 µM, 10 µM, 100 µM) (Fig. 2) for 1.5 h. This exposure induced the formation of DNA DSBs and the formation of γ-H2AX foci in a cell density dependent manner that is commensurate with total fluorescence measurements per well. The total fluorescence intensity per well of γ-H2AX foci increased with increasing etoposide concentration (Fig. 2a,b). A549 cells seeded at 10,000 or 20,000 cells per well (320 mm^2^, 96 well plate) and grown for 24 h exhibited no significant response to 10 µM etoposide but showed an increased expression of γ-H2AX after exposure to 100 µM, (**p < 0.01, ****p < 0.0001, Fig. 2a,b). A549 cells of the same seeding density grown for 48 h showed a detectable increase in γ-H2AX expression upon exposure to both 10 and 100 µM etoposide (***p < 0.001 and ****p < 0.0001, respectively). These results indicate that there is a threshold of fluorescence intensity required to detect a signal. This fluorescence intensity threshold appears to correlate with cell density and the amount of DNA damage induced. An increased cell seeding density of 30,000 or 40,000 cells per well followed by 24 h growth, and subsequent etoposide treatment (10–100 µM, 1.5 h) yielded a statistically significant increase of γ-H2AX foci as reported in aggregate fluorescence intensity per well, **p < 0.01, One-way ANOVA followed by Tukey’s multiple comparisons post-test (Fig. 2a,b). Cells grown for 48 h prior to etoposide treatment (10 µM and 100 µM) yielded a statistically significant increase in γ-H2AX foci with increasing cell seeding density. A concomitant low-level increase in background (untreated) fluorescence was observed for the higher cell seeding densities (30,000–40,000 cells) after 48 h in culture (Fig. 2b). Nuclear DNA (DAPI) fluorescence increased with cell density and was not a result of etoposide treatment (no statistical significance) (Fig. 2c, d). As cell density increased from 24 to 48 h the total DAPI fluorescence also increased (~ two-fold). At high concentrations of etoposide (100 µM) and high cell seeding density (40,000 cells), there is an apparent decrease in DAPI staining when compared to control (0 µM) or 10 µM treatment at each timepoint.

Filamentous actin (F-actin) cytoskeleton structure was also quantified using rhodamine phalloidin fluorescence as a measure of quality control to ensure consistency of cell density during immunostaining and processing. Phalloidin staining, remained consistent across all seeding densities after 24 h in culture. Labeled F-actin fluorescence however decreased after 48 h in culture most likely due to cell compaction and contraction of F-actin required to allow cell growth and expansion within each well area. No effects of etoposide on total F-actin fluorescence were determined.

### Sensitivity analysis of γ-H2AX quantitation for dose-dependent etoposide exposure

**Figure 3:**
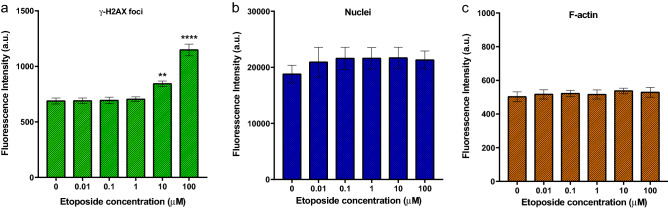
Fluorescence increases with the number of DNA double-strand breaks. A549 cells were treated with etoposide for 1.5 h, and subsequently stained for (**a**) foci for γ-H2AX phosphorylated DNA double-strand breaks, (**b**) DAPI for DNA nuclei, and (**c**) rhodamine-labeled phalloidin for F-actin. n = 12 wells per treatment and data are presented as averages ± SEM. γ-H2AX phosphorylation shows statistically significant increases for 10 μM and 100 μM compared with the no treatment control group, **P < 0.01 ****P < 0.0001, one-way ANOVA.

**Figure 4:**
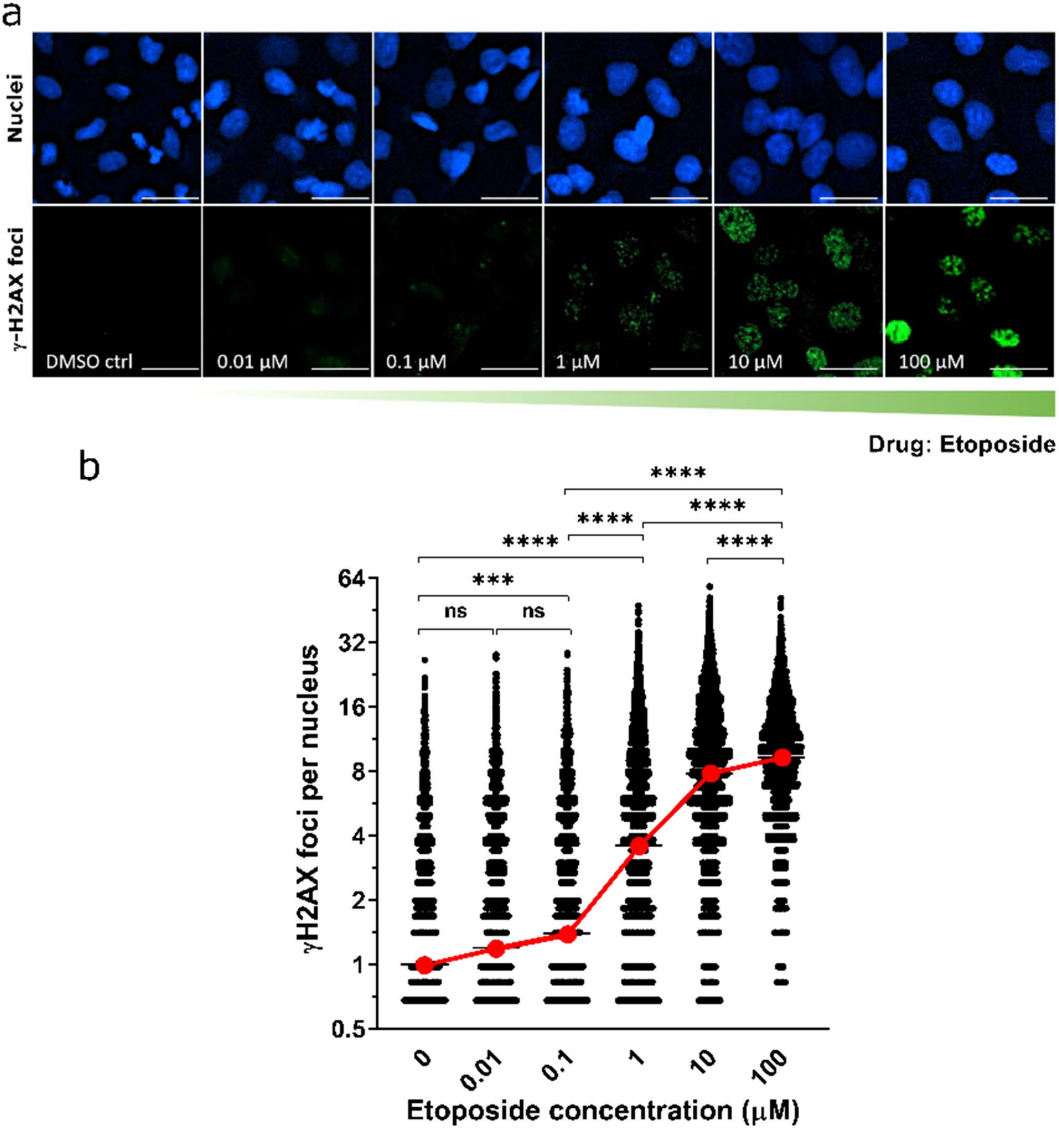
γ-H2AX foci of double-strand breaks quantified by automated spot counting algorithm. (**a**) Representative images of γ-H2AX foci in A549 cells treated with etoposide (1.5 h, 0–100 μM); γ-H2AX (green) and nuclear DNA stained with DAPI (blue). Scale bar is 20 µm. (**b**) Data summarizing Spot Count analysis and quantitation of individual γ-H2AX foci per nucleus (black) and average γ-H2AX foci per nucleus (red). The data are presented as mean ± standard deviation of 1500 nuclei per sample with four biological replicates, **P < 0.01. ***P < 0.001, ****P < 0.0001, one-way ANOVA. Average γ-H2AX for each concentration reveals a dose–response relationship (red line).

To understand the cellular impact of etoposide exposure and confirm cellular integrity, A549 cells were seeded and grown in 96-well half-area plates for DSB measurements. The γ-H2AX immunofluorescence intensity of etoposide treated A549 cells (Fig. 3) exhibited a statistically significant increase in response to higher etoposide concentrations (10 μM and 100 μM) relative to untreated control cells. This automated multi-well bulk fluorescence assay, however, did not detect changes in immunofluorescence at or below 1 μM etoposide. The increase in bulk fluorescence intensity observed at 10 and 100 µM concentrations correlates with the detection of DSBs in the cells as quantified from cellular images (Fig. 4a) and processed for γ-H2AX foci counts (Fig. 4b). The fluorescence intensity of DAPI (Fig. 3b) and phalloidin (Fig. 3c) did not change significantly with etoposide treatment.

#### Double-strand breaks quantified by algorithmic spot counting

Microscopy images of DAPI labeled nuclei and DSB formation allowed for morphological observation of γ-H2AX foci which primarily revealed a putative random distribution of individual foci in A549 cells in response to etoposide treatment (10 nM–100 μM for 1.5 h) (Fig. 4a). Using algorithmic spot counting analysis, we detected individual γ-H2AX foci of varying size at all etoposide treatment concentrations, and a less intense γ-H2AX signal distributed over the whole nucleus in regions where no dose was given (DMSO control). Etoposide induced a significant linear increase in DNA damage at 1 μM etoposide (****P < 0.0001), 10 μM etoposide (****P < 0.0001), and at 100 μM etoposide (****P < 0.0001) compared to control (DMSO vehicle), one-way ANOVA followed by Tukey multiple comparisons post-test. Further, the distribution of foci in treated cells showed one to three foci at lower etoposide concentrations (0.01–1 μM), whereas the majority of cells at higher concentrations of etoposide treatment have seven to nine foci per cell (10–100 μM) (Table 1).

**Table 1:** Normalized average foci per nucleus in response to etoposide exposure.

Etoposide concentration (µM)	Normalized average foci per nuclei	Standard error of the mean	Cells without foci	*P*-value compared to control
0	1	0.0331	2484	NA
0.01	1.19	0.0380	2555	0.2933
0.1	1.39	0.0409	2342	0.0003
1	3.58	0.0703	1516	< 0.0001
10	7.81	0.0986	631	< 0.0001
100	9.25	0.1027	316	< 0.0001

#### Pan-nuclear response is DNA damage dependent

**Figure 5:**
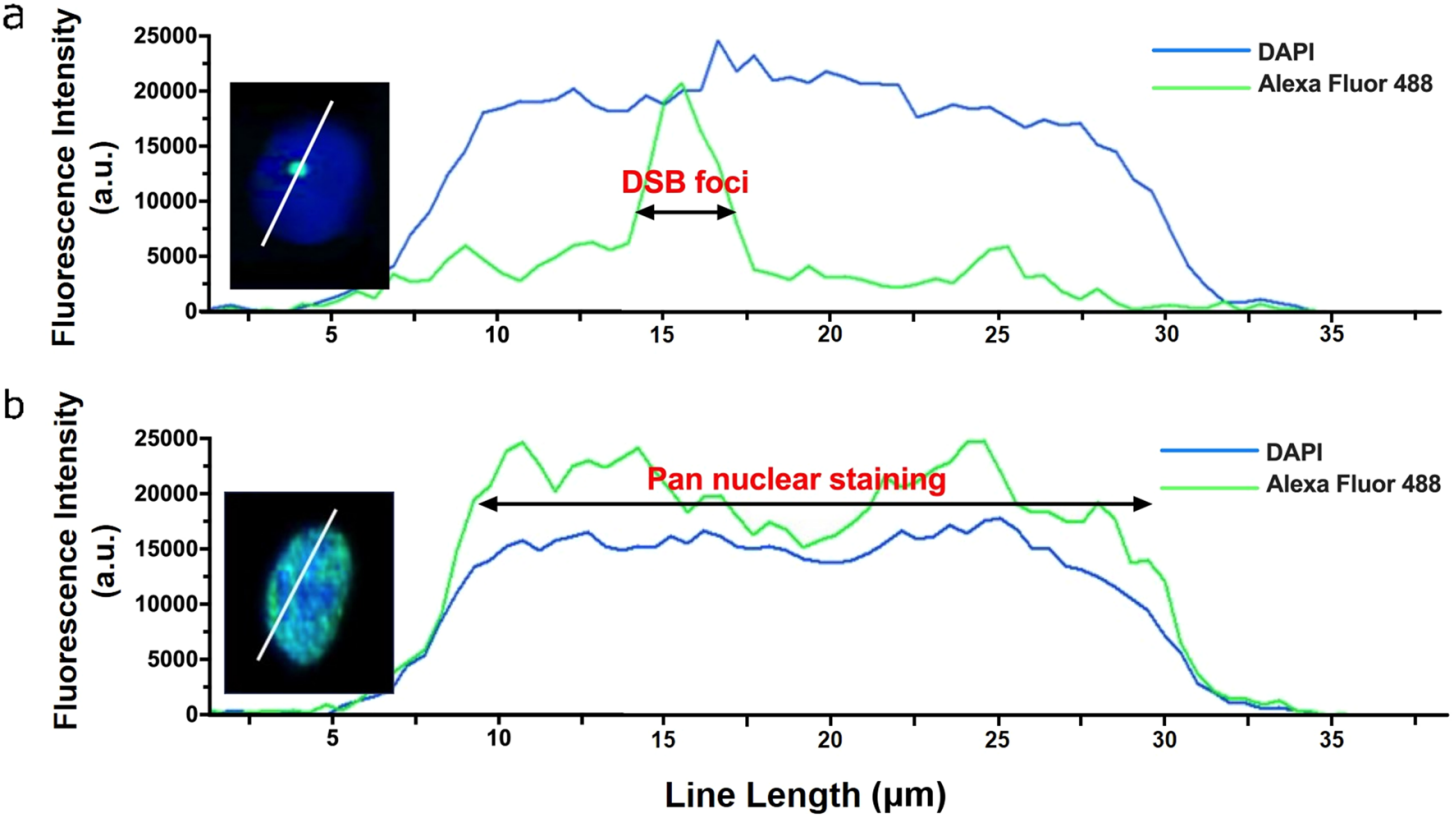
Pan-nuclear staining intensity. Representative intensity profiles of γ-H2AX (Alexa Fluor 488, green) and nuclear DNA (DAPI, blue) are shown for A549 cells. Double-strand breaks and pan-nuclear staining were assessed by measuring the length of cell nuclei that co-localized with a cross section of γ-H2AX foci when treated with (**a**) 0.1 μM etoposide and (**b**) 100 μM etoposide.

**Figure 6:**
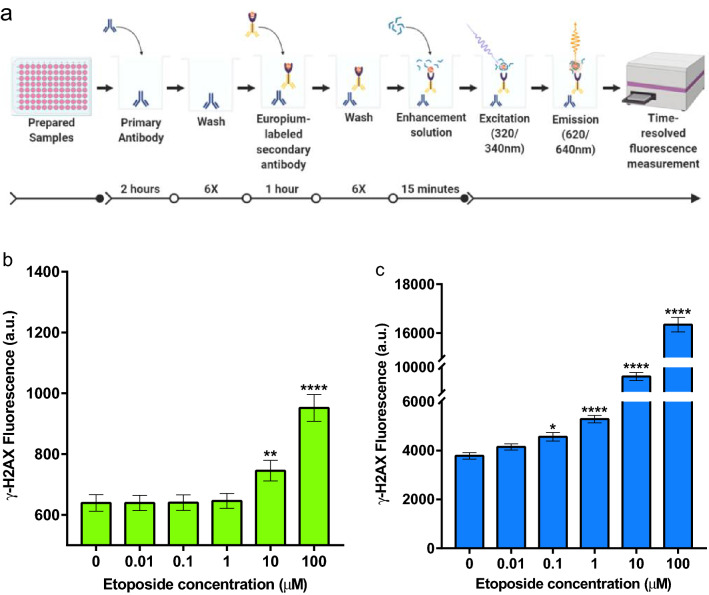
Time-resolved fluorescence (TRF) intensity significantly detects and differentiates low numbers of DNA DSBs over conventional immunofluorescence. (**a**) Schematic depicting the dissociation-enhanced lanthanide fluorescence immunoassay (DELFIA). In a 96-well plate, the prepared samples were incubated with the primary antibody for 2 h, then exposed to the europium-labeled secondary antibody for 1 h. The samples were subsequently washed 6 times and incubated with the enhancement solution for 15 min. In a plate reader, the samples were excited with a 320/340 nm wavelength, and the emission wavelengths of 620/640 nm were produced for a TRF measurement. (**b**) Micromolar detection of DNA DSBs using an anti-γ-H2AX antibody followed by an Alexa Fluor 488–labeled secondary antibody. Immunofluorescence (IF) intensity in A549 cells was measured using a standard plate reader. (**c**) Nanomolar detection of DNA DSBs using an anti-γ-H2AX antibody followed by a europium–labeled secondary antibody. Immunofluorescence intensity in A549 cells was measured using DELFIA and TRF intensity technology. Results in (**b**) and (**c**) from three separate experiments. *P < 0.05, **P < 0.01, ****P < 0.0001, one-way ANOVA followed by Tukey multiple comparisons post-test. Image in (**a**) created with Biorender.com, https://biorender.com/.

Figure 5 shows the fluorescence intensity profile comparison of A549 cells treated with 0.1 μM etoposide (Fig. 6a) and 100 μM etoposide (Fig. 6b). We observed pan-nuclear γ-H2AX after etoposide induced DNA damage to the nucleus with 100 μM treatment (Fig. 6b) with 20–30% of cells exhibiting nuclear-wide γ-H2AX. By comparison, only a single γ-H2AX foci was observed with 0.1 μM etoposide treatment (Fig. 6a), and < 1% of cells showed pan-nuclear γ-H2AX at 10 μM. No pan-nuclear H2AX phosphorylation was observed at etoposide treatments of 0.01–1 μM. DSB foci exhibit a high fluorescence intensity peak, whereas pan-nuclear γ-H2AX spans the entire nucleus as shown by line length and line convergence of DAPI and Alexa Fluor 488 (Fig. 5b).

#### DNA double-strand breaks detected at low abundance with DELFIA assay

To perform the DELFIA, A549 cells were seeded as specified in the Methods section and treated with different doses of etoposide (0.01–100 μM) for 1.5 h. The cells were fixed, permeabilized, and stained with the antibody against γ-H2AX followed by a europium-tagged secondary antibody as described in the Methods (Fig. 6a). Overall, the total fluorescence intensity measured in each well of the 96-well plate increased with increasing etoposide concentration (Fig. 6c). A linear correlation was observed between this fluorescence intensity and the dose of etoposide. This correlation exhibited two differential ranges of response; one between 0 and 1 μM (Fig. 6c) and a second between 1 and 100 μM as indicated by the need to have breaks on the Y-axis of the graph in Fig. 6c due to the high levels of fluorescence detected. A significant difference in fluorescence intensity was observed when the cells were treated with low doses of etoposide (0.1–1 μM), and an increased sensitivity of 2 orders of magnitude was observed when compared to the Alexa Fluor 488 fluorescence detection method (Fig. 6b).

## Discussion

Developing a simple and reliable method to quantify DNA DSBs is critical to improve the monitoring and assessment of DNA damage induced by hazardous agents encountered through environmental, medical, and occupational exposure. Studies have shown that the number of γ-H2AX foci directly correlates with the number of DNA DSBs in cells^17,26,27^ and not cell density. However, the type of radiation influences the size and intensity of γ-H2AX foci^14^ and whether γ-H2AX foci appear within pan-nuclear γ-H2AX staining^46,47^. In recent years, low- and high-throughput (manual and automated) methods have been developed and compared to detect and quantify γ-H2AX expression^48^. Studies have also focused on the formation of DNA DSBs in cells derived from different tissue sources^2,3,5,40–42,49,50^. In certain instances, such as mass casualty radiation scenarios manual foci counting may be preferable^19,39^ while more recent studies suggest that automated foci scoring offers the advantage of faster processing which is also key to responding to such incidents^51^. Immunofluorescence techniques and epifluorescent microscopy have been commonly used to count γ-H2AX foci with the naked eye^7,19,37,52–54^. This approach is tedious and has led to the development of high-throughput automated foci detection to quantify DNA DSBs within cell nuclei^2,3,40–42,49,55^. Some of these methods include laser-scanning cytometry^3^, infrared imaging scanners^5^, imaging flow cytometry^40^, and automated γ-H2AX immunocytochemistry^41^. Most of these methods have been proven robust and exhibit different degrees of resolution. They have been developed to handle large numbers of samples^51^ and could be optimized for other cell types. However, they require sophisticated instrumentation or costly technology not available in most standard laboratories.

Here, we have developed a simple and reliable method for multimode detection of γ-H2AX to quantify DNA DSBs in cells using a fluorescence plate reader. This assay was validated in A549 cells treated with different concentrations of etoposide to induce DNA DSBs. Fluorescence intensity of cells stained for nuclear DNA, F-actin cytoskeleton, and antibodies against γ-H2AX was measured using a multimode plate reader; secondary antibodies for this comparison were Alexa Fluor 488 or a europium-labeled antibody. Cellular counts for DAPI-labeled cellular nuclei and F-actin cytoskeleton allowed us to determine whether γ-H2AX expression was influenced by cell density (Fig. 2). This co-staining allowed us to confirm the uniformity of cell density across all wells and treatments, indicating that cell loss from sample manipulation during immunocytochemistry was negligible.

Analysis of cell nuclei fluorescence (DAPI) and F-actin cytoskeleton content allowed us to confirm the density-related contributions to etoposide treatments (Fig. 2). A drop in rhodamine-phalloidin fluorescence for F-actin was evident after 48 h of growth that most likely reflects decreased space in the wells, reduced area for expansion of cytoskeletal processes, and increased area taken up by nuclei and other cell organelles. The decrease in DAPI fluorescence at 40,000 cells and 100 µM etoposide is likely due to a high level of pan-nuclear staining for γ-H2AX, which in turn reduces the nuclear area available for uptake of the DNA stain. This decrease in DAPI uptake probably also accounts for irreparable DNA damage and breakdown of the nuclear material within the cell in response to etoposide treatment. Pan-nuclear staining of γ-H2AX is also known to have different size and intensity based on the type of radiation exposure^14,23^.Particle based irradiation response such as that induced by heavy-ion irradiation results in pan-nuclear staining that is not related to apoptosis^47^. This can also be observed when cells are under stress and when the cell is undergoing apoptosis^47^. The DAPI staining at 0–100 µM etoposide and 10,000 cells seeded is relatively similar and may reflect a lack of such staining at these concentrations.

We observed a limited detection of DNA double-strand breaks with standard immunofluorescence. A549 cells cultured for 48 h in 96-well half-area plates at (10,000 cells/half-well area) were treated with different doses of etoposide (10 nM–100 μM) for 1.5 h. This seeding density is equivalent to the 20,000 cell seeding density in Fig. 2b which reduces the number of cells required for the assay while maintaining a significant level of fluorescence detection after 48 h in culture. For these studies the cells were cultured without serum for 24 h just prior to etoposide treatment to synchronize their cell cycles. Additionally, phenol red free media was used to minimize the contribution of background cellular autofluorescence.

With the traditional Alexa Fluor 488 labeled secondary antibody, we observed a positive linear correlation between the fluorescence intensity of cells and the dose of etoposide above 1 μM (Fig. 3). When the europium-labeled secondary antibody and TRF were used to quantify the fluorescence intensity of the foci, we also observed a dose-dependent increase in fluorescence intensity (Fig. 6). The sensitivity of this assay is based on the release of the europium metal from the secondary antibody into the enhancing solution where it becomes complexed by the chelating agent. Employing TRF intensity allowed us to measure the enhanced europium-chelate fluorescence corresponding to the induction of γ-H2AX foci within cell nuclei. This resulted in a greater sensitivity of detection of DSBs induced by lower drug concentrations compared to the standard bulk fluorescent measurement (Fig. 4b) and was equivalent to the detection limits for spot counting using the Cytation 1 imager, Gen5 software, and the Spot Count algorithm. The dose-dependent increase in fluorescence intensity for TRF was significant at low doses of etoposide (0.1 µM) suggesting that TRF significantly improves detection and allows differentiation of low numbers of DNA DSBs. The many advantages of TRF include a longer life span of the fluorescence of the lanthanide chelate label compared to the traditional fluorophore Alexa Fluor 488, which gives a temporal flexibility. Additionally, the lanthanide chelate dissociates to produce a new highly fluorescent chelate, which improves the sensitivity and resolution of the assay.

A potential drawback is the fact that this assay requires the TRF software to be included in the plate reader capabilities, which is not a standard option in most plate readers. Although quantifying DNA DSBs by immunostaining using Alexa Fluor 488 did not show a comparable sensitivity to the magnitude of the lanthanide-labeled secondary antibody, this method is simpler and does not require additional plate reader capabilities. Reading bulk fluorescence at a single excitation and emission however is suitable when measuring higher amounts of DNA DSBs. Our multimode detection of DNA DSBs was complemented by more traditional γ-H2AX nuclear foci counting using the Spot Count algorithm in the BioTek Cytation 1 imager. This mode of detection showed an increase in the ability to detect foci with nanomolar concentrations of etoposide (Fig. 4). The increase in foci detected was significant for doses of etoposide greater than 100 nM. This approach allows visual detection and assessment of foci within cell nuclei (Fig. 4a) and allows for quantitative assessment of cells without any DSBs as shown in Table 1. The BioTek Spot Count algorithm and Gen5 software is required for analyzing the foci which removes some of the labor-intensive counting and allows for quantitation of larger numbers of foci and therefore streamlines the ability to assess DSBs within more cells. A limitation of the BioTek automated spot counting technique is the requirement for expensive equipment and software, although other software packages (e.g. FIJI/ImageJ, Imaris) may be able to have macros tailored to achieve a comparable result if already available.

This spot counting approach is further limited by its inability to differentiate between individual DSB foci and pan-nuclear γ-H2AX that in our study occurs mostly at higher doses of etoposide. Pan-nuclear staining is also evident when cells are distressed or going through apoptosis in the cell cycle^23,46^; however, other software applications (e.g. FIJI/ImageJ, Imaris) may be able to more readily incorporate this measure. This pan-nuclear staining might explain the plateau observed at high concentrations on the curve of the number of foci as a function of the dose of etoposide (Fig. 4). A high-throughput γ-H2AX assay was developed by Lee et al.^40^ based on imaging flow cytometry that could automatically detect and eliminate pan-nuclear γ-H2AX, hence improving the resolution of the method. This technology might be used to improve the spot counting algorithm.

In summary, we have developed multimode detection of γ-H2AX at varying sensitivity levels using standard fluorescence and TRF plate readers. This assay was used to measure the fluorescence intensity of γ-H2AX as a marker of DNA DSBs using a traditional fluorophore, Alexa Fluor 488, or a lanthanide-labeled secondary antibody in A549 cells treated with etoposide. We found that measuring the fluorescence intensity using a traditional fluorophore is a simple and reliable method to quantify DNA DSBs when the number of DNA DSBs is high. However, TRF using DELFIA is a more sensitive approach that can significantly detect and differentiate low levels of DNA DSBs. Similar analysis taking advantage of an imaging capable plate reader performed for comparison confirmed the sensitivity of this assay. The spot counting method provides a sensitivity comparable to that observed for the TRF DELFIA assay. Furthermore, it facilitates the visual assessment of the foci within the nuclei. The relative number of cells per well was also found to influence the fluorescence intensity in response to etoposide treatment. Therefore, cell density should be optimized for each cell type to ensure effective assessment of DNA DSBs independent of the γ-H2AX detection method employed. The usefulness and versatility of these multimode detection methods enables tailoring of the methodology to address specific questions and meet screening requirements necessary to develop high throughput analysis and high-resolution imaging and quantitation. These various multimode assays have the potential to improve our understanding and assessment of radiotherapies, allow for future high throughput quantification of DNA damage induced by chemicals and physical factors and screen compound libraries to identify potential protective or sensitizing agents.

## Methods

### Cell culture

Adenocarcinoma human alveolar basal epithelial cells, A549 cells, were purchased from American Type Culture Collection (ATCC, Manassas, VA, USA) and maintained as a monolayer, cultured in a 1:1 ratio of Dulbecco’s Modified Eagle Medium and Ham’s F-12 Nutrient Mixture (DMEM/F-12) (Fisher Scientific Inc., Pittsburgh, PA, USA) supplemented with 10% (v/v) fetal bovine serum (Fisher Scientific) and 1% penicillin–streptomycin. A549 cells were grown at 37 °C in a humidified incubator with 5% CO_2_. The media was changed every other day. Before etoposide treatment, cells were seeded at 10,000 cells per 50 µL in black half-area 96 well plates (Greiner Bio-One North America Inc., Monroe, NC, USA) in phenol red–free media and incubated ≥ 18 h at 37 °C in a humidified incubator with 5% CO_2_.

### Induction of DNA double-strand breaks

Etoposide (Fisher Scientific) dilutions were initially prepared in DMSO (Thermo Fisher Scientific, Waltham, MA, USA) then diluted further in phenol red-free, serum-free DMEM/F12 (Fisher Scientific) media for a final DMSO concentration of 1% (v/v). When estimating the effects of etoposide cells were cultured overnight in a phenol red-free medium without serum for 24 h. Cells were treated with etoposide (0.01–100 µM) diluted into 50 µL of serum-free DMEM/F12 media and incubated for 1.5 h. Control wells were treated with 1% (v/v) DMSO/phenol red-free, serum-free DMEM/F12 media solution. The etoposide treatments were carefully aspirated from the wells, and the cells were washed three times for 5 min each with 1 × phosphate-buffered saline (PBS) (Millipore Sigma, Burlington, MA, USA).

### Immunocytochemistry and fluorescence intensity recorded using a plate reader

Cells treated with etoposide were washed with PBS and fixed for 10 min at room temperature with 4% paraformaldehyde (Fisher Scientific). The cells were then permeabilized for 10 min at room temperature with 0.25% triton-X 100 (Fisher Scientific) in PBS. After permeabilization, the cells were washed twice with PBS (5 min each time with gentle rocking) and blocked with the blocking buffer consisting of 1% casein (Fisher Scientific) and 1% normal goat serum (Fisher Scientific) in PBS for 2 h at room temperature with gentle rocking or overnight at 4 °C. After the blocking step, the cells were incubated with the rabbit monoclonal antibody against Phospho-Histone H2AX (serine 139) (20E3) (Cell Signaling Technologies, Catalog No. 9718) at a 1:500 dilution in the blocking buffer for 2 h at room temperature with gentle rocking or overnight at 4 °C. The cells were washed twice with the wash buffer (0.05% Tween 20 in PBS, 5 min each time) and incubated for 1 h at room temperature with the secondary antibody Alexa Fluor 488 tagged anti-rabbit IgG, F(ab’)_2_ (Cell Signaling, catalog no. 4412). The cells were washed twice with the wash buffer with gentle rocking (5 min each time) and once with PBS for 5 min then dual stained for 30 min at room temperature with DAPI (ThermoFisher Scientific) and phalloidin-rhodamine conjugate (ThermoFisher Scientific, catalog no. R415) (1:1000 dilution each in PBS). The cells were washed twice with PBS for 5 min each time with gentle rocking. PBS was added to each well (100 μL/well) and the fluorescence intensity was recorded using an EnSpire multimode plate reader (PerkinElmer). For Alexa Fluor 488 the excitation was set at 490 nm and emission at 525 nm. For DAPI, the excitation was set at 358 nm and emission at 461 nm. For phalloidin-rhodamine, the excitation was set at 540 nm, and the emission was set at 565 nm.

### Immunocytochemistry for γ-H2AX assay quantified by BioTek Cytation 1 and Spot Count algorithm

The cells were fixed using a 4% (v/v) paraformaldehyde (Fisher Scientific) solution for 10 min. The cells were washed three times for 5 min each with PBS and permeabilized with 0.3% (v/v) Triton X-100 (Fisher Scientific)/PBS solution for 10 min. Cells were washed three times, for 5 min each with PBS and blocked with MAXblock Blocking Medium (Active Motif Inc., Carlsbad, CA, USA) for 2 h at room temperature and then overnight at 4 °C. The blocking agent was aspirated, and the cells were washed with the MAXwash washing medium (Active Motif) for 10 min. The primary antibody, Anti-phospho-Histone H2A.X (serine 139), clone JBW301 (MilliporeSigma), and secondary antibody, Anti-Mouse IgG (H + L), F(ab')2 fragment CF 488A (MilliporeSigma), were diluted to final concentrations of 2 and 1 µg/mL, respectively, in MAXbind Staining Medium (Active Motif). The cells were incubated with 50 µL of primary antibody for 2 h at room temperature. Cells were washed three times for 5 min each with MAXwash washing medium and incubated with 50 µL of the secondary antibody for 1 h at 37 °C. Cells were washed four times, for 5 min each with MAXwash washing medium and incubated with rhodamine-labeled phalloidin (Biotium Inc., Fremont, CA, USA) (0.0165 µM) in PBS for 30 min at room temperature. Cells were washed three times, for 5 min each then incubated with DAPI (VWR International Ltd, Lutterworth, Leicestershire, England) (0.02 µg/mL) in PBS for 1 min at room temperature. Cells were washed three times for 5 min each and incubated with PBS for data acquisition. Cells were covered in one drop (20 µL) of Mowiol 4–88 (MilliporeSigma) solution made per manufacturer's instructions to be used in data acquisition (Polysciences Inc., 2008 Data sheet 777 Mowiol 4-88).

### DELFIA and TRF to quantify DNA double-strand breaks

DELFIA uses TRF intensity technology (PerkinElmer). This technology is based on the dissociation of the lanthanide in the presence of a chelator and the formation of a fluorescent complex with longer life span and stronger signal. The cells (10,000/well) were seeded in 96 half-well plate (Greiner) and maintained for 48 h in a 5% CO_2_ incubator. They were subsequently treated with etoposide, fixed, permeabilized, and blocked as described in the immunocytochemistry section. The cells were then incubated with the primary antibody specific for γ-H2AX (Cell Signaling Technologies) at a 1:100 dilution in the DELFIA assay buffer (PerkinElmer) for 2 h at room temperature or overnight at 4 °C. The cells were subsequently washed six times with DELFIA wash buffer (PerkinElmer) (150 μL/well for 5 min with gentle rocking each time). After washing the cells, europium-labeled secondary antibody (Eu-N1-anti rabbit IgG, 50 ng in 50 µL/well) (PerkinElmer) prepared in the DELFIA assay buffer was added to the cells, and they were incubated at room temperature with gentle rocking for 1 h. The cells were washed again six times with the DELFIA wash buffer as before, and 100 μL of enhancement solution (PerkinElmer) was added to each well. The plate was shaken for 15 min at room temperature, and europium TRF was measured using the Synergy 2 plate reader (BioTek). The excitation was set at 320/340 nm, and the emission was set at 620/640 nm. All incubations at room temperature were performed with gentle rocking.

### Immunocytochemistry and fluorescence imaging with automated algorithmic foci counting to quantify DNA double-strand breaks

Biotek Cytation 1 Cell Imaging Multi-Mode Reader (BioTek Instruments Inc., Winooski, VT, USA) and Gen5 Microplate Reader and Imager Software (catalog no. GEN5, BioTek) were used to acquire data. The filter cubes used to image the cells were DAPI (Cat #1225100, BioTek), GFP (Cat #1225101, BioTek), and RFP (Cat #1225103, BioTek). The Gen5 Spot Counting Module (GEN5SPOT, BioTek) was used to quantify foci in treated nuclei. For each of the 4 biological replicates to be analyzed, 1500 nuclei were randomly selected. The mean number of spots in the no treatment control wells was normalized to 1; all other treatments were normalized to this value.

### Statistical analyses

One-way ANOVA followed by Tukey’s multiple comparisons post-test was performed using GraphPad Prism, version 8.3.1 for Windows (GraphPad Software, San Diego, CA, USA, www.graphpad.com). These tests were performed with 95% confidence intervals. *P < 0.05, **P < 0.01, ***P < 0.001, ****P < 0.0001 compared with the background value of control group for the same protein in the same type of cells.
